# Hierarchical Artificial Bee Colony Algorithm for RFID Network Planning Optimization

**DOI:** 10.1155/2014/941532

**Published:** 2014-01-23

**Authors:** Lianbo Ma, Hanning Chen, Kunyuan Hu, Yunlong Zhu

**Affiliations:** ^1^Department of Information Service & Intelligent Control, Shenyang Institute of Automation, Chinese Academy of Sciences, Faculty Office VII, Nanta Street No. 114, Dongling District, Shenyang 110016, China; ^2^University of Chinese Academy of Sciences, Beijing 100039, China

## Abstract

This paper presents a novel optimization algorithm, namely, hierarchical artificial bee colony optimization, called HABC, to tackle the radio frequency identification network planning (RNP) problem. In the proposed multilevel model, the higher-level species can be aggregated by the subpopulations from lower level. In the bottom level, each subpopulation employing the canonical ABC method searches the part-dimensional optimum in parallel, which can be constructed into a complete solution for the upper level. At the same time, the comprehensive learning method with crossover and mutation operators is applied to enhance the global search ability between species. Experiments are conducted on a set of 10 benchmark optimization problems. The results demonstrate that the proposed HABC obtains remarkable performance on most chosen benchmark functions when compared to several successful swarm intelligence and evolutionary algorithms. Then HABC is used for solving the real-world RNP problem on two instances with different scales. Simulation results show that the proposed algorithm is superior for solving RNP, in terms of optimization accuracy and computation robustness.

## 1. Introduction

In recent years, radio frequency identification (RFID) technology as a new inventory tracking technology has great promise for diversified use in many industries with numerous practical applications. Much great potential has been realized and many are being explored. In many real-world RIFD applications, such as production, logistics, supply chain management, and asset tracking, a sufficient number of readers are deployed in order to provide complete coverage of all the tags in the given area [1, 2]. Specially, over the last ten years, RFID is used to build up an “Internet of Things” (IoT), a network that connects physical things to the Internet, making it possible to access remote sensor data and to control the physical world from a distance [3]. This brings some questions in the deployment of an RFID network for the operation and management of the large-scale Internet of Things applications, such as optimal tag coverage, quality of service (QoS), and cost efficiency [4].

Due to the limited recognition range of single reader, many readers and tags need to be deployed according to some arrangement to construct the RFID system in the scenario area. This results in some necessary questions to be considered in the case of avoiding reader collision, such as (1) how many readers are needed; (2) where should the readers be placed; (3) what is the efficient parameter setting for each reader [5, 6]. In addition, considering cost-efficient for RFID system, the network should meet the items with minimum number of readers and maximum tags coverage. Thus, the RFID network planning problem (RNP) is a difficult NP problem [7, 8]. In general, we defined that the RNP aims to optimize a set of objectives (tags coverage, load balance, economic efficiency, interference between readers, etc.), by adjusting the control variables (the coordinates of the readers, the number of the readers, the antenna parameters, etc.) of the system [8].

In the past two decades, evolutionary computation (EC) and swarm intelligence (SI) techniques for solving RNP problem have gained increasing attention, such as genetic algorithms (GA) [9, 10], evolutionary strategy (ES) [11], differential evolution (DE) [12], particle swarm optimization (PSO) algorithms [6, 11, 13], and bacterial foraging algorithms (BFA) [14, 15]. Specially, in [6] we present a multispecies particle swarm optimization model for solving RNP problem, achieving a significant positioning accuracy. It is noted that some scholars propose other methods to dispose similar problems, such as bipartite graph [16, 17]. However, with the increasing number of the deployed readers and tags in the large-scale RFID deployment environment, the degree of complexity for solving the RNP optimization increases exponentially. The previous methods to solve the RNP optimization are incompetent for being prone to premature convergence [13].

A natural approach to tackle high-dimensional optimization problems is to adopt the cooperative coevolution based on divide-and-conquer strategy. An early work on a cooperative co-evolutionary algorithm (CCEA) by [18] provides a promising approach for decomposing a high-dimensional problem. Recent studies [19–23] by taking improved decomposition strategy into PSO algorithm. Inspired by these recent works, we propose a novel hierarchical coevolving scheme, extending the canonical artificial bee colony (ABC) algorithm framework from nonhierarchy to hierarchy, called hierarchical artificial bee colony algorithm (HABC). Our HABC model is inherently different from others in the following aspects.

Firstly, the cooperative coevolving approach based on divide-and-conquer strategy with random grouping technology is adopted into HABC, which enhances the local search ability (exploitation). Under this method, the high-dimensional vectors can be decomposed into smaller subcomponents which are assigned to the lower hierarchy. This method enhances the local searching ability.

Secondly, the traditional evolution operators such as crossover and mutation are applied to interaction of multispecies instead of single species enhancing the information exchange between populations. Under this new development, the neighbor bees with higher fitness can be chosen for crossover and mutation, which effectively enhances the global search and convergence to the global best solution as the dimension increases. This maintains population diversity and enhances the global search ability (exploration).

By incorporating this new degree of complexity, HABC can accommodate considerable potential for solving more complex problems. Here we provide some initial insights into this potential by evaluating HABC on both mathematical benchmark functions and a real-world RNP case, which focuses on minimizing four specific objective functions of a 30-reader RFID network and a 50-reader RFID network. The simulation results, which are compared to other state-of-the-art methods, show the superiority of the proposed algorithm.

The rest of the paper is organized as follows. In Section 2, RFID system models and the RNP problem definitions are presented.  Section 3 first gives a review of the canonical ABC algorithm and then proposes the novel HABC algorithm.  Section 4 tests the algorithm on the ten benchmarks and illustrates the results.  Section 5 describes the implementation of the proposed approach based on HABC on two instances, Cd100 and Rd100, and the results of simulation are analyzed. Finally, Section 6 outlines the conclusions.

## 2. Problem Formulation on RNP

**Figure 1:**
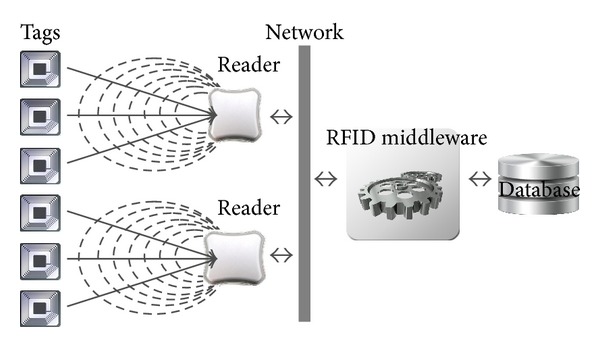
RFID system.

An RFID system consists of four types of important components (see Figure 1): (1) RFID tags, each placed on an object and consists of a microchip and an embedded antenna containing a unique identity, which is called Electronic Product Code (EPC); (2) RFID readers, each has more than one antenna and is responsible to send and receive data to and from the tag via radio frequency waves; (3) RFID middleware, which manages readers and filters and formats the RFID raw tag data; (4) RFID database, which records RFID raw tag data that contain information such as reading time, location, and tag EPC. In this section, a mathematical optimization model for the RNP problem based on RFID middleware is proposed.

The model is constructed from several different aspects. The deployment region of hotspots is supposed to be a two-dimension square domain. The tags here are passive and are based on the Class-1 Generation 2 UHF standard specification [6, 9, 12]. It means that they can only be powered by radio frequency energy from readers. The proposed RNP model aims to improve the QoS of RFID networks by optimizing the objects including coverage, interference, load balance, and aggregate efficiency via regulating the parameters of RFID networks, including the number, location, and radiated power of readers. Generally the problem is formulated as follows.

### 2.1. Optimal Tag Coverage (*f*_*c*_)

The first objective function represents the level of coverage, which is the most important in an RFID system. In this paper, if the radio signal received at a tag is higher than the threshold *δ* = −10 dBm, the communication between reader and tag can be established. Then the function is formulated as the sum of the difference between the desired power level *δ* and the actual received power *P*_*i*_^*j*^ of each tag *i* in the interrogation region of reader *j*:

(1) $$\begin{array}{c} \mathrm{Min}f_{\text{c}}=\sum_{i\in \text{TS}}^{}\;\sum_{j\in {\text{RS}}_{i}}^{}\left(P_{i}^{j}-\delta \right), \end{array}$$

where TS and RS are the tag and reader set that are deployed in the working area respectively, and RS_*i*_ represents the set of readers which has the tag *i* in its interrogation region. This object function ensures that the received power *P*_*i*_^*j*^ at the tag *i* from the reader *j* in RS_*i*_, which is mainly determined by the relative distance and radiated power of the reader *j*, is higher than the threshold *δ*, which guarantees that the tag is activated. That is, by regulating the locations and radiated power of the readers, the optimization algorithm should locate the RFID readers close to the regions where the desired coverage level is higher, while the areas requiring lower coverage are taken into account by the proper radiated power increases of the readers.

### 2.2. Reader Interference (*f*_*i*_)

Reader collision mainly occurs in a dense reader environment, where several readers try to interrogate tags at the same time in the same area. This results in an unacceptable level of misreads. The main feature of our approach is that the interference is not solved by traditional ways, such as frequency assignment [24] and reader scheduling [6], but in a more precautionary way. This objective function is formulated as

(2) $$\begin{array}{c} \mathrm{Min}f_{i}=\sum_{k\in \text{RS}}^{}\;\sum_{i\in {\text{TS}}_{k}}^{}\left(\delta -\left(P_{i}^{k}-\sum_{j\in {\text{RS}}_{i}}^{j\neq k}P_{i}^{j}\right)\right), \end{array}$$

where TS_*k*_ is the tag set in the interrogation region of reader *k*. For each tag *i*, this objective considers all the readers except the best one as interfering sources. That is, by changing reader positions and powers according to this functional the algorithm tries to locate the readers far from each other to reduce the interference.

### 2.3. Economic Efficiency (*f*_*e*_)

This aspect could be approached from various points of view. For example, due to the stochastic noise, multipath effect, and attenuation in the propagation channel, readers should be located closely to the center of tags in the hotspots. From this perspective, this objective can be reached by weighing the distances of each center of tag clusters from its best served reader. Here we employ *K*-means clustering algorithm to find the tag cluster. It can be defined below as follows:

(3) $$\begin{array}{c} \mathrm{Min}f_{e}=\sum_{k\in \text{RS}}^{}\mathrm{dist}\left(R_{k},\theta_{k}\right), \end{array}$$

where dist⁡() is the distance between the *k*th reader and the *k*th tag center and *θ*_*k*_ and *R*_*k*_ are the position of *k*th cluster center and its best served reader, respectively. In this way the algorithm tries to reduce the distance from the readers to the elements with high tag densities.

### 2.4. Load Balance (*f*_*b*_)

A network with a homogeneous distribution of reader cost can give a better performance than an unbalanced configuration [25]. Thus, in large-scale RFID system, the set of tags to be monitored needs to be properly balanced among all readers. This objective function is formulated as

(4) $$\begin{array}{c} \mathrm{Min}f_{l}=\prod_{k\in \text{RS}}\left(\frac{C_{k}^{\max}}{C_{k}}\right), \end{array}$$

where *C*_*k*_ is the assigned tags number to reader *k* and *C*_*k*_^max⁡^ is the maximum number of tags which can be read by the reader *k* in unit time. It should be noticed that the *C*_*k*_^max⁡^ takes different values according to the different types of readers used in the network. This object aims to minimize the variance of load conditions by changing the locations and radiated power of readers.

### 2.5. Combined Measure (*f*_*m*_)

In this paper, the overall optimal solution for RNP is represented by a linear combination of the four objective functions:

(5) Minimize  fc=∑i=14wififimax⁡;w1+w2+w3+w4=1, wi>0,

where *f*_*i*_ is the objective function for the *i*th requirement normalized to its maximum value *f*_*i*max⁡_; the normalization is necessary because these four objectives represent nonhomogeneous quantities and are very different in values.

### 2.6. Objective Constraint

All the tags in working area must be covered by a reader. This constraint can be formally expressed by the following formula:

(6) s.t.Pij−δ≥0 ∀i∈TS, j∈RSi,∑k∈RSλik≥1 ∀i∈TS,

where *λ*_*i*_^*k*^ is a binary variable in which *λ*_*i*_^*k*^ = 1 if the reader *k* ∈ RS_*i*_; otherwise *λ*_*i*_^*k*^ = 0. So this constraint can maintain the power efficiency of network and ensure a complete coverage deployment.

## 3. Hierarchical Artificial Bee Colony Algorithm

### 3.1. Canonical Artificial Bee Algorithm

The ABC algorithm is a relatively new SI algorithm by simulating the foraging behaviors of honey bee swarm, initially proposed by Karaboga and further developed by Basturk and Akay [26–28]. In ABC, the colony of artificial bees contains three groups of individuals, namely, the employed, onlookers and scouts bees. Employed bees exploit the specific food sources and give the quality information to the onlooker bees. Onlooker bees receive information about the food sources and choose a food source to exploit depending on the quality information. The employed bee whose food source has been abandoned becomes a scout and starts to search for a new food source. The fundamental mathematic representations are listed as follows.

In initialization phase, the algorithm generates a group of food sources that correspond to the solutions in the search space. The food sources are produced randomly within the range of the boundaries of the variables. Consider

(7) $$\begin{array}{c} x_{i,j}=x_{j}^{\min}+\text{rand}\left(0,1\right)\left(x_{j}^{\max}-x_{j}^{\min}\right), \end{array}$$

where *i* = 1,2,…, SN and *j* = 1,2,…, *D*. SN is the number of food sources and equals to half of the colony size. *D* is the dimension of the problem, representing the number of parameters to be optimized. *x*_*j*_^min⁡^ and *x*_*j*_^max⁡^ are lower and upper bounds of the *j*th parameter, respectively. Additional, counters which store the numbers of trials of each bee are set to 0 in this phase.

In the employed bees' phase, each employed bee is sent to the food source in its memory and finds a neighboring food source. The neighboring food source is produced according to (8) as follows:

(8) $$\begin{array}{c} v_{i,j}=x_{i,j}+\varphi \left(x_{i,j}-x_{k,j}\right), \end{array}$$

where *k* is a randomly selected food source different from neighbor *i*. *j* is a randomly selected dimension. *ϕ* is a random number which is uniformly distributed in range [−1,1]. The new food source *v* is determined by changing one dimension on *x*. If the value in this dimension produced by this operation exceeds its predetermined boundaries, it will set to be the boundaries.

The new food source is then evaluated. A greedy selection is applied on the original food source and the new one. The better one will be kept in the memory. The trials counter of this food will be reset to zero if the food source is improved; otherwise, its value will be incremented by one.

In the onlooker bees' phase, the onlookers receive the information of the food sources shared by employed bees. Then they will choose a food source to exploit depending on a probability related to the nectar amount of the food source (fitness values of the solution). That is to say, there may be more than one onlooker bee choosing the same food source if the source has a higher fitness. The probability is calculated according to (9) as follows:

(9) $$\begin{array}{c} P_{i}=\frac{{\text{fitness}}_{i}}{\sum_{j=1}^{\text{SN}}{\text{fitness}}_{j}}. \end{array}$$

After food sources have been chosen, each onlooker bee finds a new food source in its neighborhood following (8), just like the employed bee does. A greedy selection is also applied on the new and original food sources.

In scout bees' phase, if a food source has not been improved for a predetermined cycle, which is a control parameter called “limit,” the food source is abandoned and the bee becomes a scout bee. A new food source will be produced randomly in the search space using (7), as in the case of initialization phase.

The employed, onlooker, and scout bees' phase will recycle until the termination condition is met. The best food source which presents the best solution is then outputted. The pseudo-code of original ABC algorithm is illustrated in Algorithm 1.

### 3.2. The Hierarchical Artificial Bee Colony Algorithm

The HABC integrates a two-level hierarchical co-evolution scheme inspired by the concept and main ideas of multipopulation co-evolution strategy and cross and mutation operations. The flowchart of the HABC is shown in Figure 1. It includes four important strategy approaches: variables decomposing approach, random grouping of variables, background vector calculating approach, and cross and mutation operation, which is presented as follows.

#### 3.2.1. Hierarchical Multipopulation Optimization Model

As described in Section 3.1, we can see that the new food source is produced by a perturbation coming from a random single dimension in a randomly chosen bee. This causes that an individual may have discovered some good dimensions, while the other individuals that follow this bee are likely to choose worse vectors in *D* dimensions and abandon the good ones. On the other hand, when solving complex problems, the canonical ABC algorithm based on single population suffers from the following drawback: as a population evolves, all individuals suffer premature convergence to the local optimum in the first generations. This leads to low population diversity and adaptation stagnation in successive generations.

**Figure 2:**
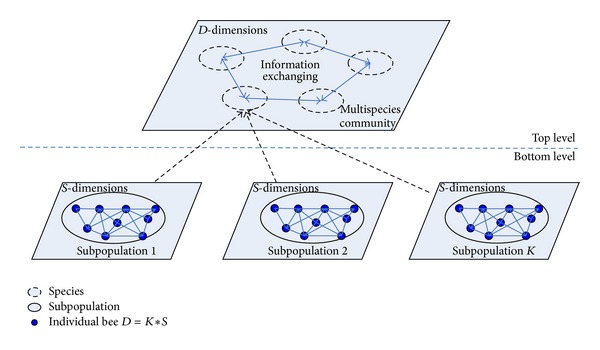
Hierarchical optimization model.

Hence, the HABC contains two levels, namely, the bottom level and top level, to balance exploring and exploiting ability. In Figure 2, in the bottom level, with the variables decomposing strategy, each subpopulation employs the canonical ABC method to search the part-dimensional optimum in parallel. That is, in each iteration, *K* subpopulations in the bottom level generate *K* best solutions, which are constructed into a complete solution species that update to the top level. In the top level, the multispecies community adopts the information exchange mechanism based on crossover operator, by which each species can learn from its neighborhoods in a specific topology. The vectors decomposing strategy and information exchange (i.e., crossover operator) can be described in detail as follows.

#### 3.2.2. Variables Decomposing Approach

The purpose of this approach is to obtain finer local search in single dimensions inspired by the divide-and-conquer approach. Notice that two aspects must be analyzed: (1) how to decompose the whole solution vector, and (2) how to calculate the fitness of each individual of each subpopulation. The detailed procedure solving those is presented as follows.

**Step 1**

The simplest grouping method is permitting a *D*-dimensional vector to be split into *K* subcomponents, each corresponding to a subpopulation of *s*-dimensions, with *M* individuals (where *N* = *K*∗*s*). The *j*th subpopulation is denoted as *P*_*j*_, *j* ∈ [1,…, *K*].

**Step 2**

Construct complete evolving solution *G*_best_ which is the concatenation of the best subcomponents' solutions *P*_*j*_ by the following:

(10) $$\begin{array}{c} G_{\text{best}}=\left(P_{1}\cdot g,P_{2}\cdot g,P_{j}\cdot g,\ldots ,P_{K}\cdot g\right), \end{array}$$

where *P*_*j*_ · *g* represents the personal best solution of the *j*th subpopulation.

**Step 3**

For each component *P*_*j*_, *j* ∈ [1,…, *K*], do the following:

- *at employed bees' phase*, for each individual *X*_*i*_, *i* ∈ [1,…, *M*]; replace the *i*th component of the *G*_best_ by using the *i*th component of individual *X*_*i*_; calculate the new solution fitness: *f*(new *G*_best_(*P*_1_ · *g*, *P*_2_ · *g*, *X*_*i*_, …, *P*_*k*_ · *g*)). If *f*(new *G*_best_) < *f*(*G*_best_), then *G*_best_ is replaced by new *G*_best_.
- update *X*_*i*_ positions by using (8);
- at onlooker Bees' Phase, repeat (a)-(b).

**Step 4**

Memorize the best solution achieved so far; compare the best solution with *G*_best_ and memorize the better one.

Under this method, high-dimensional objective function vectors can be decomposed into smaller subcomponents, which are evolving separately. This multipopulations parallel processing approach enhances the local searching ability.

*Random Grouping of Variables*. To increase the probability of two interacting variables allocated to the same subcomponent, without assuming any prior knowledge of the problem, according to the random grouping of variables proposed by [20, 21], we adopt the similar random grouping scheme by dynamically changing group size. For example, for a problem of 100 dimensions, we can define that

(11) $$\begin{array}{c} G=\left\{2,5,10,20,100\right\},\\ K\subset G. \end{array}$$

Here, if we randomly decompose the *D*-dimensional object vector into *K* subcomponents in each iteration (i.e., we construct each of the *K* subcomponents by randomly selecting *S*-dimensions from the *D*-dimensional object vector), the probability of placing two interacting variables into the same subcomponent becomes higher over an increasing number of iterations.

#### 3.2.3. The Information Exchange Mechanism Based on Crossover Operator between Multispecies

**Figure 3:**
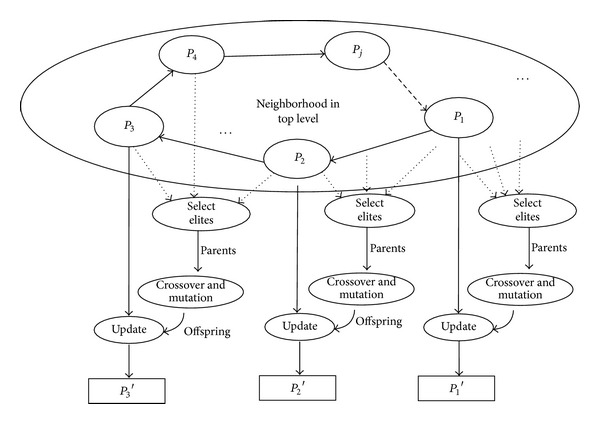
The information exchange mechanism based on crossover operator.

In the top level, we adopt crossover operator with a specific topology to enhance the information exchange between species, in which each species *P*_*j*_ can learn from its symbiotic partner in the neighborhood. The key operations of this crossover procedure are described in Figure 3.

**Step 1 (select elites to the best-performing list (BPL))**

First, a set of competent individuals from current species *P*_*j*_'s neighborhood (i.e., ring topology) are selected to construct the best-performing list (BPL) with higher fitness has larger probability to be selected. The size of BPL is equal to the number of current species *P*_*j*_. These individuals of BPL are regarded as elites. The selection operation tries to mimic the maturing phenomenon in nature, where the generated offspring will become more suitable to the environment by using these elites as parents.

**Step 2 (crossover and mutation between species)**

To produce well-performing individuals, parents are selected from the BPL's elites only for the crossover operation. To select parents effectively, the tournament selection scheme is used, in which two enhanced elites are selected randomly, and their fitness values are compared to select the elites, and the one with better fitness value is regarded as parent. Then another parent is selected in the same way. Two offsprings are created by performing crossover on the selected parents. This paper adopts the arithmetic crossover method: the offspring is produced by

(12) $$\begin{array}{c} S_{\text{new}}=\text{rand}\left(0,1\right)\times \text{parent}\;1+\text{rand}\left(0,1\right)\times \text{parent}\;2, \end{array}$$

where *S*_new_ is the newly produced offspring and parent 1 and parent 2 are randomly selected from BPL.

**Step 3 (update with greedy selection strategy)**

Not all current species are replaced by the elites from BPL; we set a selecting rate CR to determine the replaced individuals. Assuming that species size of *P*_*j*_ is *M*, then the replaced individuals number is *M*∗CR. For the selected individual *S*_*j*_, the newly produced offspring *S*_new_ is then compared with *S*_*j*_, applying a greedy selection mechanism, in which the better one is remained. We can choose four selecting approaches: selecting the best individuals (i.e., *M*∗CR individuals), a medium level of individuals, the worst individuals, and random individuals. Hence, there are several HABC variants according to different selecting approach. Here, we choose the simplest approach (i.e., selecting the worst individuals) to be replaced.

## 4. Benchmark Test

In the experimental studies, according to the no free lunch (NFL) theorem [29], a set of 10 benchmark functions, which are listed in Appendix A, are employed to fully evaluate the performance of the HABC algorithm without a biased conclusion towards some chosen problems. In order to compare the different algorithms fairly, we decide to use the number of function evaluations (FEs) as a time measure substituting the number of iterations due to the reason that the algorithms do differing amounts of work in their inner loops.

### 4.1. Experimental Settings

Experiments are conducted with six variants of HABC according to the different CR values. The proposed HABC is compared with six successful EA and SI algorithms: artificial bee colony algorithm (ABC) [26], cooperative co-evolutionary algorithm (CCEA) [18], canonical PSO with constriction factor (PSO) [30], cooperative PSO (CPSO) [19], genetic algorithm with elitism (EGA) [31], and covariance matrix adaptation evolution strategy (CMA-ES) [32].

ABC is a recently developed SI paradigm simulating foraging behavior of bees [26]. CCEA is the earliest cooperative coevolutionary algorithm which applied the divide-and-conquer approach by Potter and de Jong [18]. CPSO is a cooperative PSO model, cooperatively coevolving multiple PSO subpopulations [19]. EGA is the classical genetic algorithm with elitist selection scheme [31]; the underlying idea of CMA-ES is to gather information about successful search steps and to use that information to modify the covariance matrix of the mutation distribution in a goal directed, derandomized fashion [32].

In all experiments in this section, the values of the common parameters used in each algorithm such as population size and total generation number are chosen to be the same. Population size is set as 50 and the maximum evaluation number is set as 100000. For the fifteen continuous testing functions used in this paper, the dimensions are all set as 100*D*.

All the control parameters for the EA and SI algorithms are set to be default of their original literatures: initialization conditions of CMA-ES are the same as in [32], and the number of offspring candidate solutions generated per time step is *λ* = 4*μ*; the limit parameter of ABC is set to be SN × *D*, where *D* is the dimension of the problem and SN is the number of employed bees. The split factor for CCEA and CPSO is equal to the dimensions [18, 19]. For canonical PSO and CPSO, the learning rates *c*_1_ and *c*_2_ are both set as 2.05 and the constriction factor *χ* = 0.729. For EGA, intermediate crossover rate of 0.8, Gaussian mutation rate of 0.01, and the global elite operation with a rate of 0.06 are adopted [31]. The parameter setting for all algorithms is listed in Table 1. For the proposed HABC, the species number *N*, split factor *K* (i.e., the subpopulation number), and the selection rate CR should be tuned firstly in the next section.

### 4.2. Sensitivity in Relation to Parameters of HABC

#### 4.2.1. Effects of Species Number *N*

The number of species of HABC in top level needs to be tuned. Three benchmarks-Sphere, Rosenbrock, and Schwefel are used to investigate the impact of this parameter. The selection rate can be set as CR = 1, and the involved benchmark functions (i.e., Sphere, Rosenbrock, and Schwefel) are run 50 times. From Figure 5, we can observe that when *N* increased, we obtained faster convergence velocity and better results on all test functions. However, it can be observed that the performance improvement is not evident when *N* > 10 for most test functions. Thus, in our experiments, the species number *N* for HABC is set at 10 for all test functions.

#### 4.2.2. Choices of CR

The basic benchmark functions (*f*_1_–*f*_5_) are adopted to evaluate the performance of HABC variants with different CRs. Form Table 2, we can find that HABC variant with CR equal to 1 performed best on four functions among all five functions, while CR equal to 0.05 got the best result on one function. According to the results with different CRs, we chose CR equal to 1 as an optimal value for the next experiments.

#### 4.2.3. Effects of Dynamically Changing Group Size *K*

Obviously, the choice of value for split factor *K* (i.e., subpopulation number) had a significant impact on the performance of the proposed algorithm. In order to vary *K* during a run, we defined *S* = {2,5, 10,50,100} for 100*D* function optimization and the split factor *K* is chosen uniformly at random from a set *S*. Then, the variant of HABC with dynamically changing *K* is compared with that with fixed split number on four benchmark functions for 50 sample runs. From the results listed in Table 3, we can observe that the performance is sensitive to the predefined *K* value. HABC, using a dynamically changing *K* value, consistently gave a better performance than the other variants except *f*_2_. Moreover, in most real-world problems, we do not have any prior knowledge about the optimal *s* value, so the random grouping scheme can be a suitable solution.

### 4.3. Comparing HABC with Other State-of-the-Art Algorithms on CEC Benchmark Functions

To verify the effectiveness of the proposed algorithm, CEC2005 functions (*f*_6_–*f*_10_) are adopted to evaluate the performance of all algorithms. According to Section 4.2, we ensure the following optimal parameter setting of HABC: CR = 1, *N* = 10, and *K* ⊂ *S*, in comparison with CCEA, CPSO, CMA-ES, ABC, PSO, and EGA algorithms.  Table 4 showed the experimental results (i.e., the mean and standard deviations of the error values (*f*(*x*) − *f*(x*)) values found in 50 sample runs) for each algorithm on *f*_6_–*f*_10_.  Figure 6 shows the search progress of the average values for all algorithms.

The mentioned five shifted and rotated functions *f*_6_–*f*_10_ are regarded as the most difficult functions to optimize. HABC outperformed CMA-ES on three out of all the five functions, except *f*_7_ and *f*_10_. HABC can find the global optimum for *f*_6_ within 10000 FEs, because the adopted variables decomposing approach can enhance the local search, which is a key contributing factor to handle high-dimensional problem. On the other hand, CMA-ES converges extremely fast. However, it either converged very well or tended to become stagnant very quickly, especially on the multimodal functions (*f*_9_,*f*_10_). From the rank values presented in Table 4, the performance of all algorithms tested here is ordered as HABC > CMA-ES > ABC > CCEA > CPSO > PSO > EGA.

## 5. RFID Network Planning Based-HABC Algorithm

In this section, the details of proposed approach to solve the RNP problem are described.

### 5.1. Solution Representation of RNP Problem

In this work, the task of RFID network planning is to deploy several RFID readers in the working area in order to achieve five goals described in Section 4.1.  Figure 7 shows an example of a working area containing 100 RFID tags and 1 RFID reader, where the following three decision variables are chosen in this work:

 
*X*: the *x*-axis coordinate value of the RFID reader;
 
*Y*: the *y*-axis coordinate value of the RFID reader;
 
*P*: the read range (i.e., radiate power level) of the RFID reader.

These variables can be encoded into solution's representation shown in Figure 7. We employ a representation in which each solution is characterized by a *D* = 3*N*_*r*_ (*N*_*r*_ is the total number of readers are that deployed in the network) dimensional real number vector. In the representation, 2*N*_*r*_ dimensions indicate the coordinates of the readers in the 2-dimensional working area, and the other 1*N*_*r*_ dimensions denote the interrogation range of each reader (which is determined by the radiated power).

### 5.2. Implementation of the HABC Algorithm for the RNP Problem

**Figure 4:**
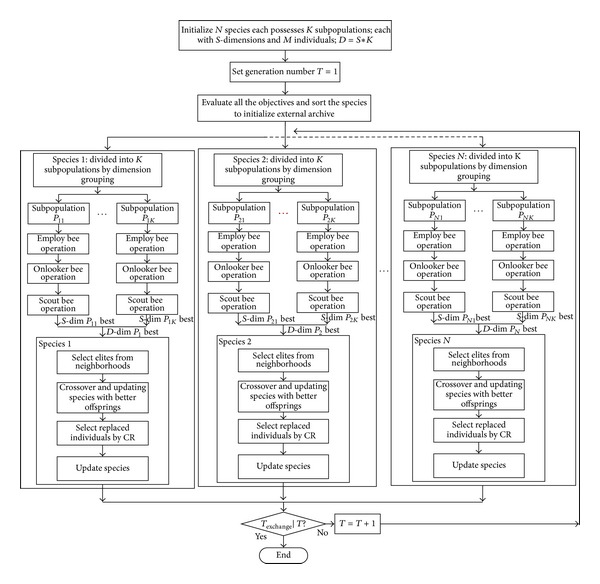
Flowchart of the HABC algorithm.

**Figure 5:**
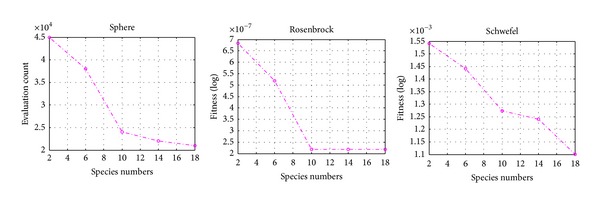
Results on test functions with different species number *N*.

**Figure 6:**
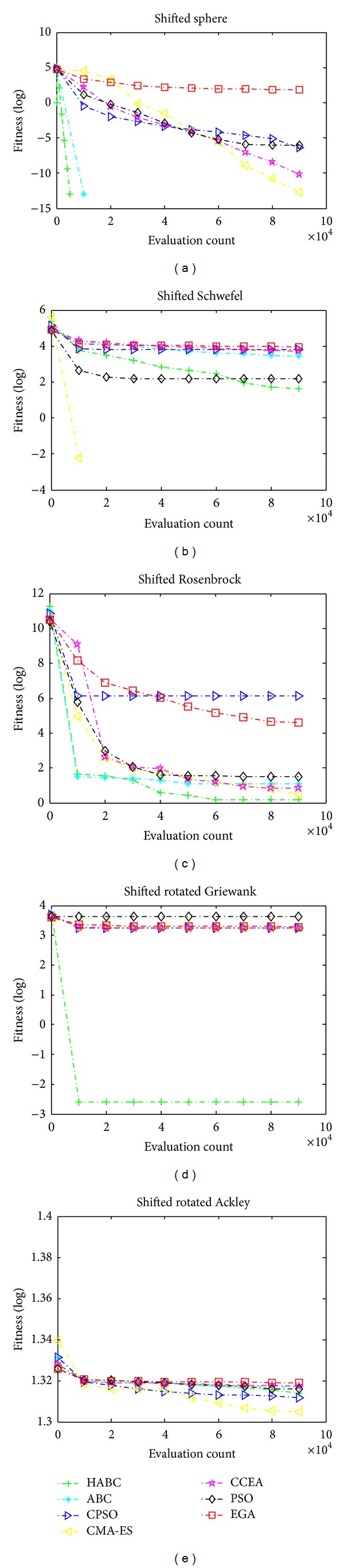
The median convergence results on 100*D* CEC functions. (a) Shifted sphere function; (b) shifted Schwefel's function; (c) shifted Rosenbrock's function; (d) shifted rotated Griewank's function; (e) shifted rotated Ackley's function.

**Figure 7:**
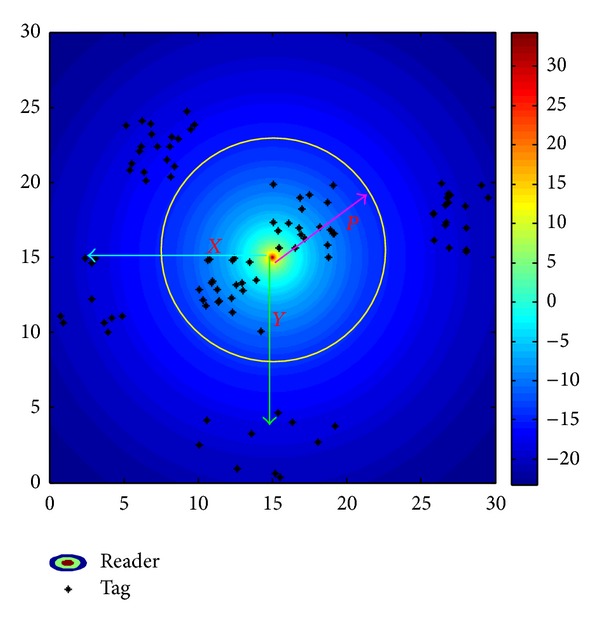
Example of a 30 m × 30 m working area with one RFID reader and 100 tags. The radiated power level (dBm) marked on the right diagram sidebar and the yellow cycle represents the read range of the RFID reader.

To apply the HABC algorithms to solve the RNP problem, the following steps should be taken and repeated (Figure 4).

**Step 1 (RFID deployment parameters initialization)**

The deployment parameters consist of reader control variables and RFID networks topology. The former include the adjustable radiated power range, the corresponding recognition scope—the distance up to which tag can be read by the reader, the interference range—the distance within which reader collision mainly occurs.

The networks topology includes the shape and dimension of the region, the number of the RFID tags to be used, the tag distribution (i.e., the tag position) in the working area, and the tag power threshold—the minimum tag received power level under which the communication between reader and tag can be established.

**Step 2 (encoding)**

Readers' variables consisting of the position and radiated power range should be encoded into the algorithm individual's representation as Table 5. The boundary limit of solution is defined by the networks topology.

**Step 3 (population generation)**

Produce the initial HABC population. Initialize *N* species; each is divided into *K* subpopulation. Each subpopulation *P*_*ij*_ possesses *M* individuals, where *i* ∈ [1,…, *N*], *j* ∈ [1,…, *K*]. Then *N* × *M* × *K*  (*N* ≥ 2, *M* ≥ 2, *K* ≥ 1) individuals based on *D*-dimensional objective should be randomly generated as shown in Figure 17, where *x*_*ijks*_  (*i* ∈ [1,…, *M*], *j* ∈ [1,…, *S*], *k* ∈ [1,…, *K*], *s* ∈ [1 … *N*],  *S* = *D*/*K*) is the position of the *j*th state variable in the *i*th individual of *k*th subpopulation of the *s*th species. *K* is the group number by dividing *D*-dimensions into *S*-dimension. Emphasize that the group number *K* is dynamically changed by random grouping approach by (11).

Notice that, each individual is characterized has a dimension *D* equal to 3*N* (*N* is the number of used RFID readers, *N* = 10 in this case), in which 2*N* dimensionalities for the coordinates of reader positions, and 1*N* dimensionalities for radiated powers of each reader.

**Step 4 (construct complete evolving solution *G*_best_)**

*G*_best_ is the concatenation of the best subcomponents' solutions *P*_*ij*_.

Set cycle⁡ = 1.

**Step 5 (optimization procedure)**

Loop over each species *P*_*i*_, *i* ∈ [1,…, *N*]: for each subpopulation *P*_*ij*_, *j* ∈ [1,…, *K*], do the following:

1. fitness calculation:
2. for each individual *X*_*i*_, *i* ∈ [1,…, *M*], replace the *i*th component of the *G*_best_ by using the *i*th component of *X*_*i*_.
3. Calculate the new solution fitness: *f*(new *G*_best_(*P*_1_ · *g*, *P*_2_ · *g*, *X*_*i*_,…, *P*_*k*_ · *g*)) using (1)–(5).
4. If *f*(new *G*_best_) < *f*(*G*_best_), then *G*_best_ is replaced by new *G*_best_;
5. update *X*_*iks*_ positions using (8);
6. memorize the best solution achieved so far, compare the best solution with *G*_best_ and memorize the better one;
7. select elites to the best-performing list (BPL) from current species' neighborhood.
8. crossover and mutation between species by (12);
9. update species with greedy selection strategy;
10. cycle⁡ = cycle⁡+1.

**Step 6 (termination condition)**

If the cycle is greater than limited value, stop the procedure; otherwise, go to Step 5.

### 5.3. Simulation Configurations

**Figure 8:**
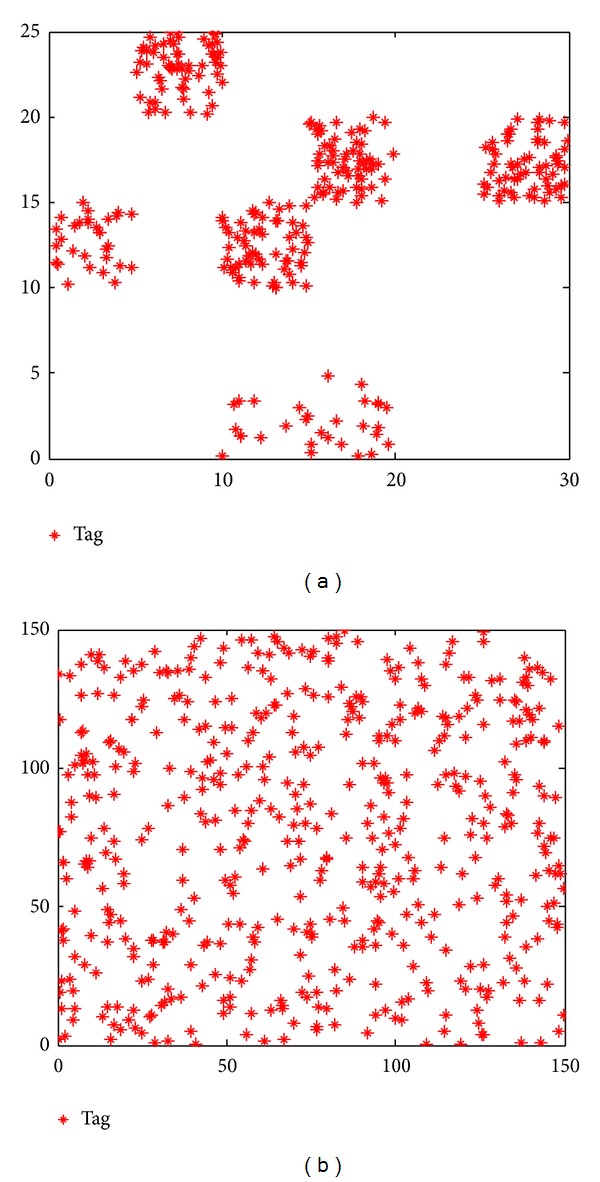
The test working area tags deployment: (a) cluster deploying with 100 tags (Cd100); (c) randomly deploying with 500 tags (Rd500).

The readers used here are mobile and the tags are passive. According to the references [6, 33–37], the related RFID readers' parameters can be set as in Table 6. Here the interrogation range according to the reader radiated power is computed as in [38]. The proposed algorithm is evaluated against two different RNP instances, namely, Cd100 and Rd500. The instance of Cd100 is tested on a 30 m × 30 m working space with 100 clustered distributed tags. Another instance, namely, Rd500, contains 500 randomly distributed tags in a 150 m × 150 m working space (shown in Figure 8). In this experiment, the parameters setting for HABC, ABC, PSO, EGA, and CCEA can be the same as in Section 4.1. Especially, the PS^2^O algorithm proposed by us in [6] as an effective approach for solving RNP, is employed to compare with the proposed approach using HABC. For PS^2^O, the number of swarms and swarm size can be set by *n* = 10, *m* = 5. The constriction factor is used with *χ* = 0.729, and then the learning rates *c*_1_ = *c*_2_ = *c*_3_ = 1.3667 [6].

### 5.4. Results of the RNP with Cd100

In this section, an RNP instance, called Cd100, in which 10 readers are deployed in the 30 m × 30 m working space with 100 clustered distributed tags, is employed, which can be considered as a continuous optimization problem with 30 dimensions, shown in Figure 8(a). All algorithms are firstly tested on the four objective functions (*f*_*c*_, *f*_*i*_, *f*_*e*_, and *f*_*l*_), respectively. In the single objective optimization, the results are providing an optimal solution for a single objective that does not take account of the others. After that, the test of the combined objective function *f*_*m*_ is implemented. The weighted coefficients used in this instance are set as *w*_1_ = 0.35, *w*_2_ = 0.15, *w*_3_ = 0.2, and *w*_4_ = 0.3, which also can be varied according to the demand of different network. The results consisting of the best, worst, mean, and standard deviation of the optimal solutions over 50 sample runs are listed in Table 7.

From Table 7, it is observed that the HABC can get better results in most proposed objective functions in comparison to other algorithms (PS^2^O, EGA, CCEA, and PSO), except *f*_*i*_. Particularly, the good performance in the combined objective function *f*_*m*_, suggests that the proposed approach using HABC outperforms the other algorithms in optimizing the models presented in this paper.

**Figure 9:**
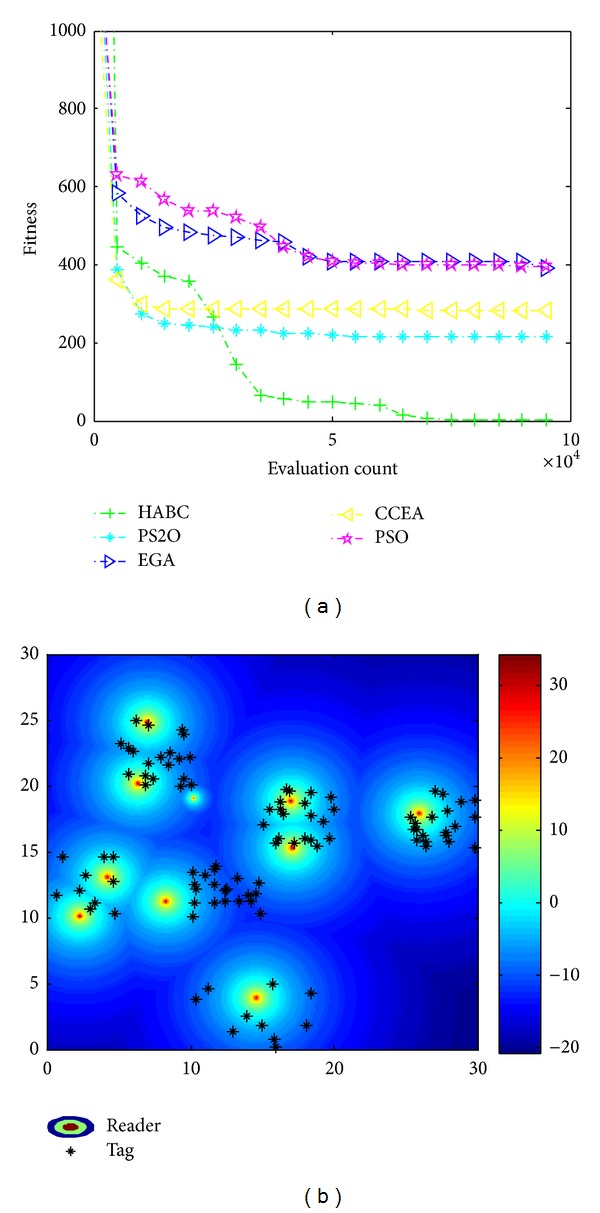
Results only consider tag coverage. (a) Convergence process; (b) reader location and received power distribution.

Figure 9 illustrates the result only considering the coverage of readers.  Figure 9(a) gives the convergence process of the average values obtained by HABC and other algorithms over 50 sample runs for the objective function *f*_*c*_. The corresponding reader locations and the distribution of their radiated power optimized by HABC are shown in Figure 9(b). In this case, according to the demand of higher tag coverage, the algorithms adjust the power and balance the deployment of readers in the working area. From Figure 9(a), the HABC has a faster convergence and gets better results than the other three algorithms. From Figure 9(b), it is obviously observed that the HABC can schedule with the reader network with higher tag coverage.

**Figure 10:**
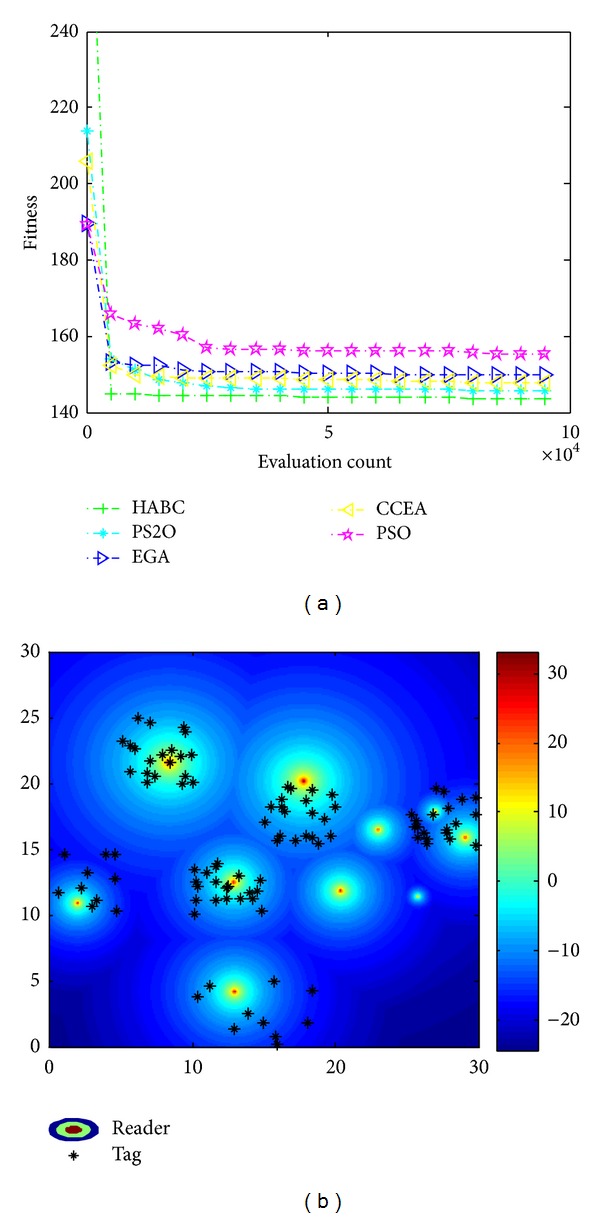
Results only consider interference. (a) Convergence process; (b) reader location and received power distribution.

**Figure 11:**
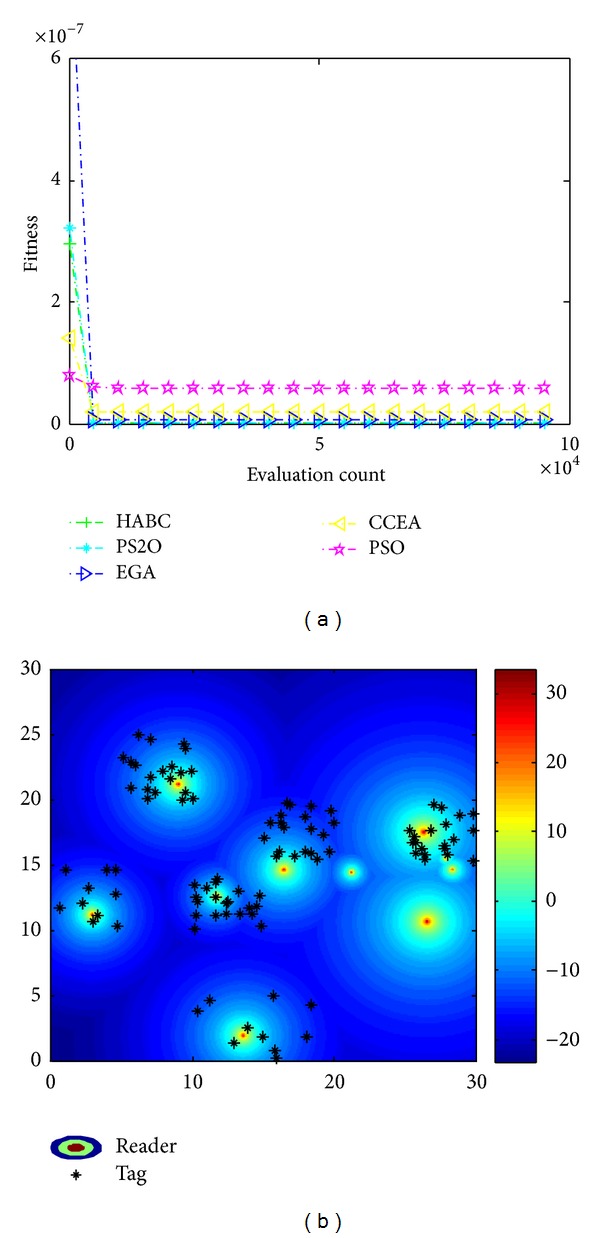
Results only consider economic efficiency. (a) Convergence process; (b) reader location and received power distribution.

**Figure 12:**
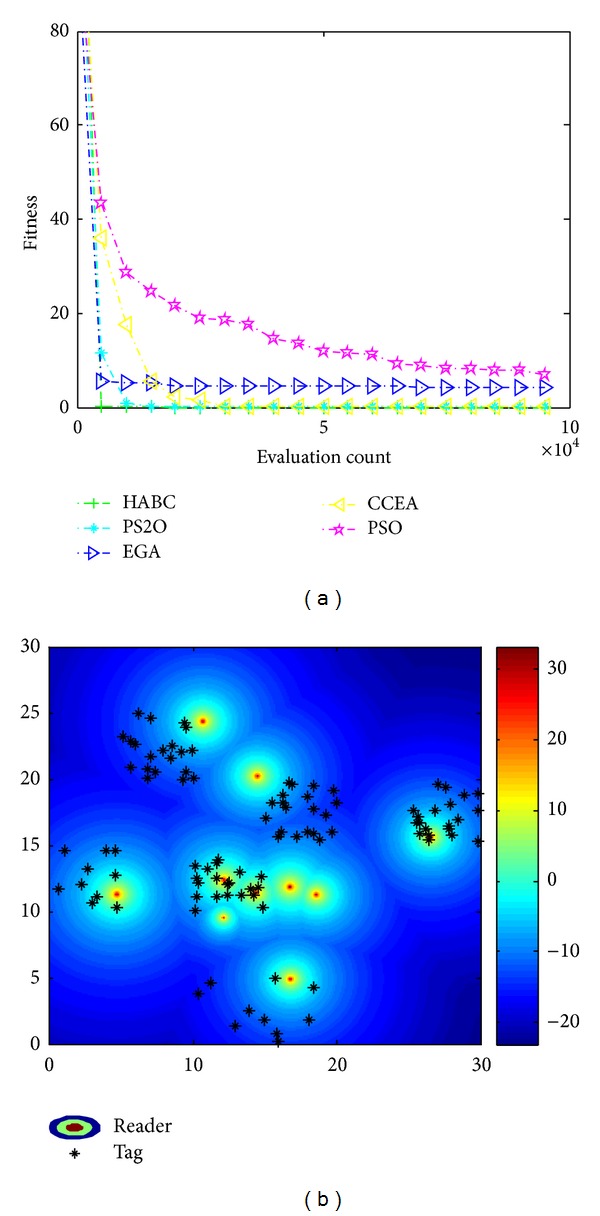
Results only consider load balance. (a) Convergence process; (b) reader location and received power distribution.

**Figure 13:**
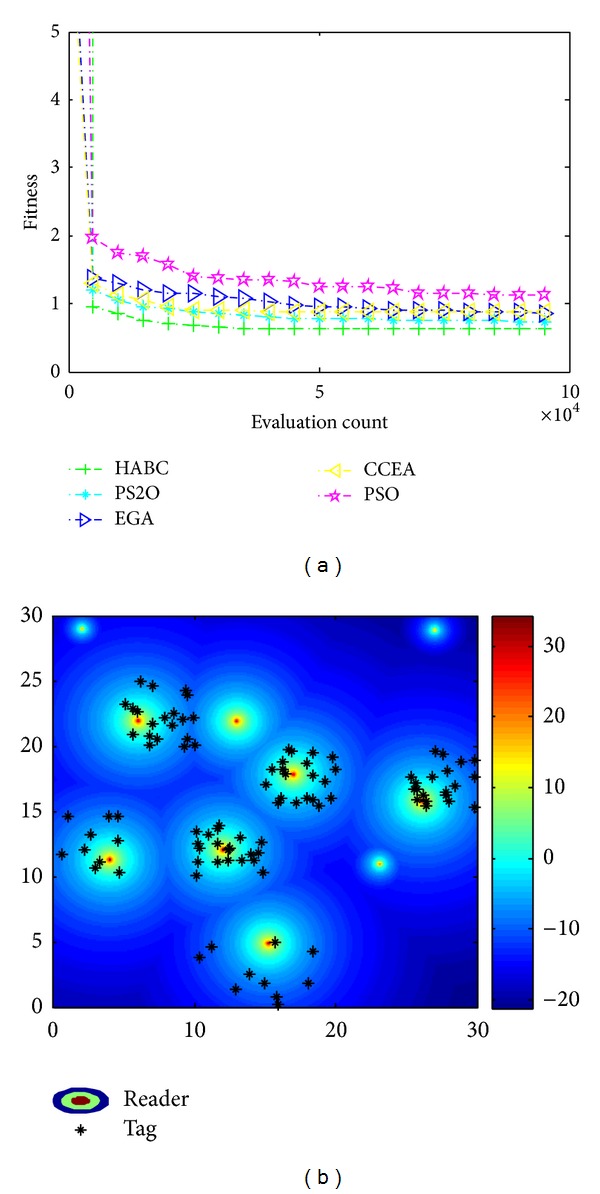
Results of the combined measurement. (a) Convergence process; (b) reader location and received power distribution.

**Figure 14:**
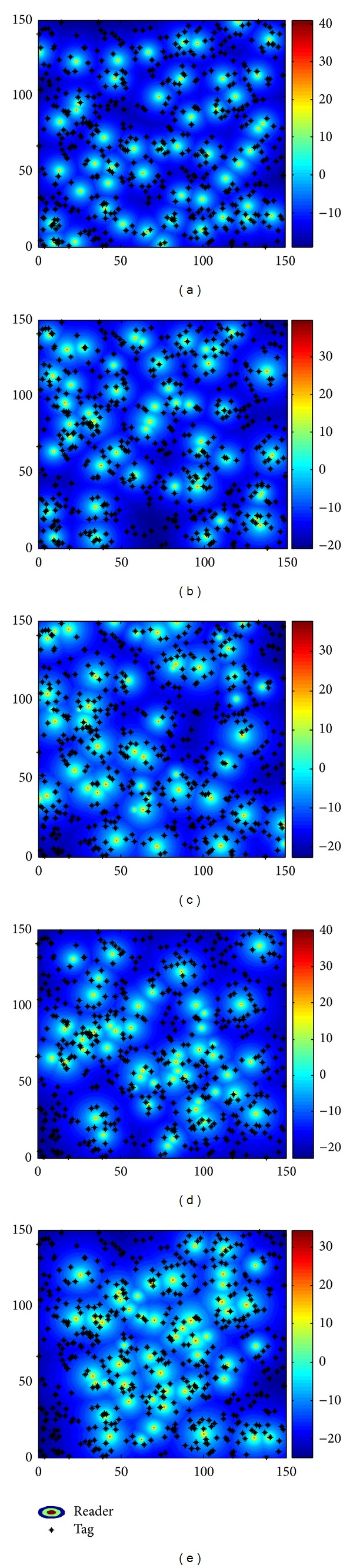
The results of all algorithms on Cd500 with the combined objective function *f*_*m*_. (a) Result of HABC; (b) result of PS^2^O; (c) result of EGA; (d) result of CCEA; (e) result of PSO.

Note that the visual results of the RNP with other four single objects have similar trends, as shown in Figures 10–14.  Figure 10 illustrates the result implemented by all algorithms with only considering the interference between readers. In this optimization mode, the algorithms aim to maintain sufficient distances between RFID readers. We can observe from Figure 10(b) that network operating condition deteriorates, without guaranteeing complete tag coverage rate while the objective function *f*_*i*_ is perfectly optimized, because the readers are optimized to move away from each other and thus located far from high traffic areas for the purpose of minimizing the reader interference.

Similarly, Figures 11 and 12 show the results considering economic efficiency and load balance, respectively. In the optimization mode with only considering economic efficiency, the algorithms aim to locate the readers close to the cluster center of the tag dense areas. From Figures 11(a) and 11(b), it is clear to see that the HABC algorithm gets significant superiority in the convergence and accuracy of solutions. From Figure 12, HABC outperforms all the other algorithms in the optimization mode with balancing the number of tags served by each reader and radiated power of the reader.

Figure 13(a) shows the convergence process of all algorithms with the combined objective function *f*_*m*_ when all the requirements are considered. We can observe from Figure 13(b) that HABC still finds the best solution, which is a reasonable compromise between different demands. Moreover, the convergence process of the five objective functions has a similar varying tendency, proving that the proposed HABC has faster convergence rate than other algorithms.

### 5.5. Results of the RNP with Rd500

Apparently, with the increasing number of the deployed readers and tags in the working area, the complexity of solving the RNP problem increases exponentially. Therefore, to further verify the efficiency of the proposed algorithm, the instance with larger scales, namely, Rd500, is also employed: 50 readers are deployed in the 150 m × 150 m working space with 500 tags that are randomly distributed, which can be considered as a continuous optimization problem with 150 dimensions, shown in Figure 8(b). Similar to the test in Section 5.4, the proposed approach using HABC and other algorithms is firstly tested with the four single objects presented in (1)–(5), respectively. Then the test of the combined objective function is implemented. The weighted coefficients are the same as in Section 5.4. All the testing results are listed in Table 8 over 50 sample runs. As shown in Table 8, the results demonstrate that the proposed approach using HABC obtains superior solutions consisting of best, worst, and mean values to those of other algorithms on all the five objective functions, better than the performance of HABC tested on Cd100 instance in which HABC outperforms other algorithms on three of five objective functions. As expected, by employing the decomposing strategy of HABC, the networks problem can be divided into several smaller ones to reduce the computational complexity, achieving better results.

**Figure 15:**
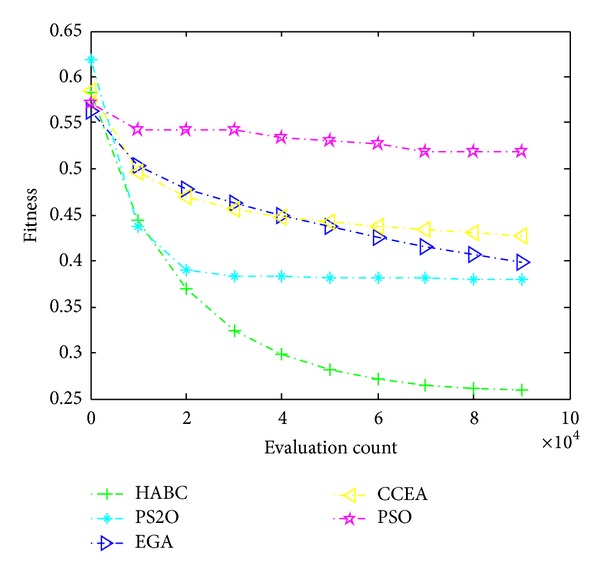
The convergence process of all algorithms with the combined objective function *f*_*m*_ on Cd500.

The reader locations and the distribution of their radiated power optimized by all algorithms are shown in Figures 14(a)–14(e), respectively. The results in Figure 14 demonstrate that the proposed approach using HABC can ensure a reasonable deployment in the aspects of maximizing the tag coverage, minimizing the interference between readers, minimizing the distances between readers and tags cluster centers, optimizing the load balance, and outperforming the compared algorithms.  Figure 15 shows that the HABC still has a faster convergence progress than other algorithms in handling larger-scale problem.

### 5.6. Algorithm Complexity Analysis

**Figure 16:**
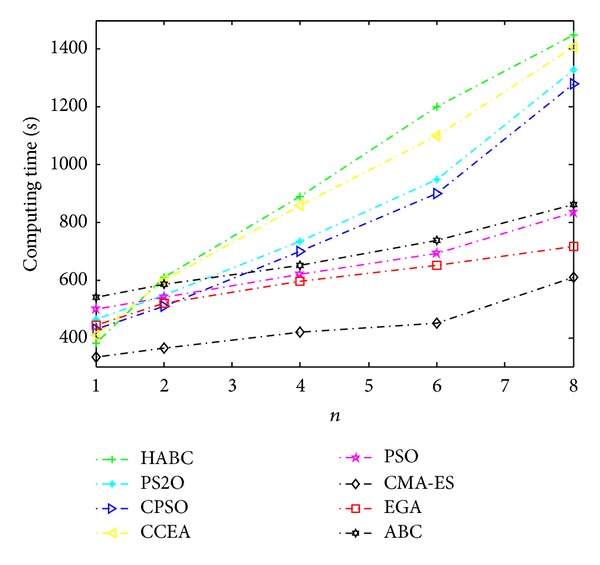
Computing time of all algorithms on different RNP problems.

**Figure 17:**
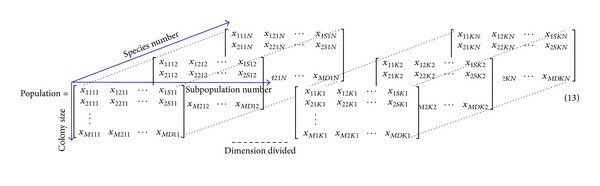


Algorithm complexity analysis is presented briefly as follows. If we assume that the computation cost of one individual in the HABC is Cost_*a*, the cost of the crossover operator is Cost_*c* and the total computation cost of HABC for one generation is *N*∗*K*∗*M*∗Cost_*a* + *N*∗Cost_*c*. However, because the heuristic algorithms used in this paper cannot ensure comprehensive convergence, it is very difficult to give a brief analysis in terms of time for all algorithms. By directly evaluating the algorithmic time response on different objective functions, the average computing time in 30 sample runs of all algorithms is given in Figure 16. From the results in Figure 16, it is observed that the HABC takes the most computing time in all compared algorithms and the time increasing rate of it is the highest one. This can explain that the multi-population cooperative co-evolution strategy integrated by HABC enhanced the local search ability at cost of increasing the computation amount. In summary, it is concluded that compared with other algorithms, the CMOABC requires more computing time to achieve better results.

## 6. Conclusion

In this paper, we present an optimization model for planning the positions and radiated power setting of readers in the RFID network. The four nonlinear RNP objective functions are formulated by considering tag coverage, reader interference, economic efficiency, and network load balance as the primary requirements of the real-world RFID system. And the combined measure is also given so that the multiple objectives can be optimized simultaneously.

Finally, in order to solve the RNP model, a novel hierarchical artificial colony algorithm, called HABC, is proposed by extending single artificial bee colony (ABC) algorithm to hierarchical and cooperative mode by combining the multi-population cooperative co-evolution approach based on vector decomposing strategy and the comprehensive learning method. Results obtained from the proposed approach have been compared with those obtained by ABC, PSO, CPSO, EGA, CMA_ES, and CCEA. The experiment results show that for all the test functions the HABC gets significant superiority to the other six algorithms. HABC is then employed to solve the real-world RNP problem on two different-scale instances, namely, Cd100 and Rd500. The simulation studies show that the HABC remarkably outperforms other algorithms. Especially, in tackling larger-scale RNP problem (i.e., Rd500 instance), the HABC performs more effectively, indicating that the HABC is more suitable for solving high-dimension RNP problem.

## Highlights

(i) Hierarchical structure and cooperative coevolution ensure the local search ability of the HABC.
(ii) The concept of crossover and mutation operator is used in HABC.
(iii) The HABC is proved to have better performance than the PSO, ABC, CPSO, EGA, CCEA, and CMA_ES on benchmarks.
(iv) An RNP optimization model is proposed based on novel HABC algorithm.
(v) HABC obtains better optimal solutions than PS^2^O, PSO, CCEA, and EGA on Cd100 and Rd500 instances.
(vi) HABC has considerable potential for solving high-dimensional optimization problems.

## Figures and Tables

**Algorithm 1:**
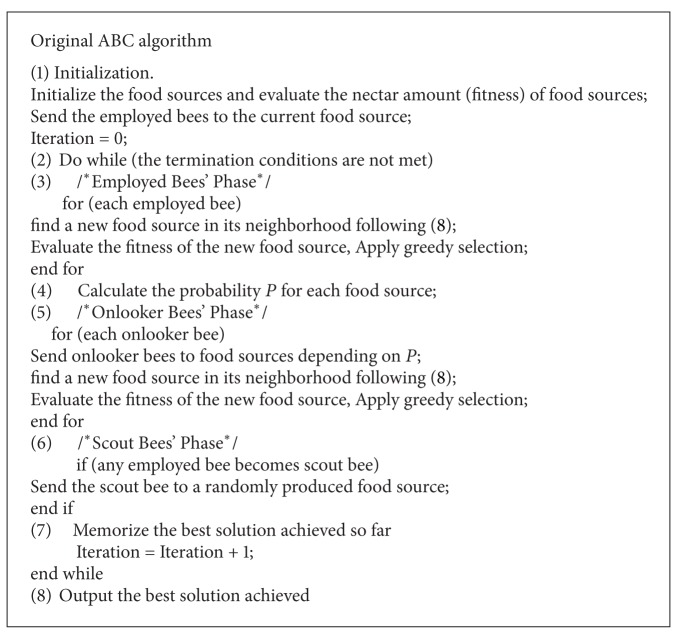
Pseudocode of the original ABC algorithm.

**Table 1:** Parameters setting for all algorithms.

Type	HABC	ABC	CPSO	PSO	CCEA	EGA	CMA-ES
*N*	10	NA	NA	NA	NA	NA	NA
*M*	5	50	5	50	5	50	50
*K*	{2, 5, 10, 50}	NA	50	NA	50	NA	NA
CR	1	NA	NA	NA	NA	NA	NA
*χ*	NA	NA	0.729	0.729	NA	NA	NA
*c*1	NA	NA	2.05	2	NA	NA	NA
*c*2	NA	NA	2.05	2	NA	NA	NA
*μ/* *c*_*σ*_ */d*_*σ*_ */c*_*c*_ */c*_cov⁡_	NA	NA	NA	NA	NA	NA	12/0.1/20/0.12/0.08
Crossover rate	NA	NA	NA	NA	0.6	0.8	NA
Mutation rate	NA	NA	NA	NA	0.02	0.01	NA

**Table 2:** Performance of HABC on 100*D* benchmark functions *f*_1_–*f*_5_ with different CRs. In bold are the best results. Note that values indicated in brackets denoted to function evaluations (FEs) are used to obtain the optimal value.

CR		0.05	0.1	0.2	0.4	0.6	1
*f*_1_	Mean	1.69*e* − 109	3.74*e* − 215	4.25*e* − 290	0 (64800)	0 (56000)	**0 (36000)**
	Std	3.79*e* − 109	1.79*e* − 201	3.19*e* − 200	4.79*e* − 210	6.59*e* − 200	3.06*e* − 200

*f*_2_	Mean	2.17*e* − 004	1.16*e* − 006	1.08*e* − 005	5.50*e* − 005	4.26*e* − 006	4.35**e** − 007
	Std	4.07*e* − 004	2.1*e* − 006	8.15*e* − 006	12.30	6.38*e* − 006	5.69**e** − 007

*f*_3_	Mean	2.36*e* − 101	2.99*e* − 194	1.71*e* − 267	0 (69700)	0 (49800)	**0 (38000)**
	Std	4.74*e* − 101	8.79*e* − 109	3.01*e* − 109	9.89*e* − 109	7.87*e* − 100	1.61*e* − 110

*f*_4_	Mean	1.93**e** − 028	4.46*e* − 028	4.97*e* − 028	5.14*e* − 026	7.62*e* − 25	7.87*e* − 022
	Std	2.03**e** − 028	8.01*e* − 028	3.44*e* − 028	5.77*e* − 026	5.55*e* − 025	1.34*e* − 021

*f*_5_	Mean	0 (20000)	0 (13500)	0 (7900)	0 (6860)	0 (4400)	**0 (3100)**
	Std	1.21*e* − 200	1.79*e* − 209	3.71*e* − 209	4.75*e* − 209	6.01*e* − 209	1.21*e* − 209

**Table 3:** Performance of HABC on 100*D* benchmark functions *f*_1_–*f*_4_ with the different grouping number *K*. In bold are the best results. Note that values indicated in brackets denoted to function evaluations (FEs) are used to obtain the optimal value.

Func.		*K* ⊂ *S*	*K* = 5	*K* = 10	*K* = 50	*K* = 100
*f*_1_	Mean	**0 (91000)**	**0 (45000)**	2.00*e* − 080	3.70*e* − 043	6.56*e* − 042
	Std	**0**	**0**	1.03*e* − 001	6.31*e* − 043	1.27*e* − 041

*f*_2_	Mean	1.10*e* − 014	1.47	1.56	6.50*e* − 001	1.61**e** − 022
	Std	1.37*e* − 014	3.12*e* − 001	2.47*e* − 001	1.13	1.38**e** − 022

*f*_3_	Mean	**0**	**0**	7.25*e* − 014	5.39*e* − 013	8.22*e* − 013
	Std	**0**	**0**	1.45*e* − 013	2.32*e* − 013	1.33*e* − 013

*f*_4_	Mean	1.70**e** − 003	7.69*e* − 002	4.45*e* − 002	3.23*e* + 002	1.99*e* + 002
	Std	1.10**e** − 003	1.11*e* − 001	2.71*e* − 002	5.37*e* + 002	3.94*e* + 002

**Table 4:** Performance of all algorithms on 100*D* benchmark functions *f*_6_–*f*_10_. In bold are the best results.

Func.		HABC	ABC	CPSO	CMA-ES	CCEA	PSO	EGA
*f*_6_	Mean	**0**	1.13*e* − 013	3.37*e* + 002	5.68*e* − 014	2.27*e* − 012	2.06*e* + 001	7.12*e* + 001
	Std	1.12*e* − 021	4.92*e* − 014	5.84*e* + 002	4.92*e* − 014	1.58*e* − 012	4.61*e* + 001	1.66*e* + 001
	Rank	**1**	3	7	2	4	5	6

*f*_7_	Mean	3.91*e* + 001	2.10*e* + 003	6.14*e* + 003	**0**	4.50*e* + 003	1.44*e* + 002	8.93*e* + 003
	Std	4.96*e* + 001	1.52*e* + 002	1.17*e* + 004	5.68**e** − 014	1.32*e* + 003	1.10*e* + 002	5.18*e* + 003
	Rank	2	4	6	**1**	5	3	7

*f*_8_	Mean	1.39	1.21*e* + 001	1.33*e* + 006	1.59	6.74	3.20*e* + 001	2.66*e* + 004
	Std	8.90**e** − 001	7.09	1.21*e* + 006	2.18	4.47	3.31*e* + 001	2.11*e* + 004
	Rank	**1**	4	7	2	3	5	6

*f*_9_	Mean	2.46**e** − 003	1.72*e* + 003	1.73*e* + 003	1.72*e* + 003	1.72*e* + 003	4.13*e* + 003	1.92*e* + 003
	Std	5.51**e** − 003	2.39*e* − 011	3.30	3.02*e* − 001	4.41*e* − 008	4.23*e* + 002	4.62*e* + 001
	Rank	**1**	3	5	2	3	7	6

*f*_10_	Mean	2.06*e* + 001	2.07*e* + 001	2.04*e* + 001	2.01**e** + 001	2.07*e* + 001	2.05*e* + 001	2.08*e* + 001
	Std	5.22*e* − 002	1.01*e* − 001	6.10*e* − 002	3.82**e** − 001	4.04*e* − 002	1.40*e* − 001	1.69*e* − 002
	Rank	3	5	2	**1**	5	4	7

**Total Rank**		**9**	**19**	**27**	**8**	**20**	**24**	**32**

**Table 5:** Representation of an individual solution *i*. Nr is the maximum number of RFID readers that are deployed in the working area.

	Reader 1 variables			Reader 2 variables			…	Reader *N*_*r*_ variables		
Solution *i*	*X*_*i*_^1^	*Y*_*i*_^1^	*P*_*i*_^1^	*X*_*i*_^2^	*Y*_*i*_^2^	*P*_*i*_^2^	…	*X*_*i*_^*N*_*r*_^	*Y*_*i*_^*N*_*r*_^	*P*_*i*_^*N*_*r*_^

**Table 6:** The example for Cd100, Rd500 and Rd500.

	Reader Specification		Topology Specification	
Cd100	Reader number	10	Dimension	30 m × 30 m
	Radiated power	0.1–2 watt	Tag number	100
	Interrogation range	3-4 m	Tag distribution	Uniform
	Interference range	3.5–4.5 m	Tag power threshold	−10 dBm

Rd500	Reader number	50	Dimension	150 m × 150 m
	Radiated power	0.1–2 watt	Tag number	500
	Interrogation range	3-4 m	Tag distribution	Uniform
	Interference range	3.5–4.5 m	Tag power threshold	−10 dBm

**Table 7:** Performance of all algorithms on RNP with Cd100.

Objective Func.		HABC	PS^2^O	EGA	CCEA	PSO
*f*_*c*_	Best	218.2832	**216.4532**	359.0912	233.3948	390.0938
	Worst	**247.1265**	268.2351	411.9823	334.3984	400.8675
	Mean	**221.0934**	241.4512	389.4652	282.9834	395.8343
	Std	**80.3842**	102.4323	91.0912	83.2387	130.3323

*f*_*i*_	Best	144.0932	**142.0934**	147.3454	145.8723	150.0923
	Worst	146.8812	**145.6421**	152.4325	156.0912	163.6433
	Mean	145.9823	**144.4326**	149.6783	148.8542	155.0933
	Std	**3.8721**	5.9564	9.8123	14.9812	26.0232

*f*_*e*_	Best	2.1583**e** − 015	2.5612*e* − 014	2.6234*e* − 01	2.1678*e* − 014	2.6563
	Worst	2.8923**e** − 015	3.3412*e* − 014	3.6973*e* − 01	4.09823*e* − 014	3.4938
	Mean	2.5812**e** − 015	5.5434*e* − 014	3.1034*e* − 01	2.7023*e* − 014	3.1276
	Std	2.58923**e** − 011	5.1654*e* − 014	3.8712*e* − 01	4.9898*e* − 011	2.6512

*f*_*l*_	Best	5.2401**e** − 0020	3.0934*e* − 013	2.9845*e* − 01	6.3412*e* − 009	3.3675
	Worst	3.2467**e** − 0019	16.883	0.0045	4.3658*e* − 008	17.0912
	Mean	1.3935**e** − 0019	3.9812	0.0009	2.4519*e* − 008	9.9814
	Std	6.6902**e** − 0020	5.7652	0.0022	7.0914*e* − 009	4.6575

*f*_*m*_	Best	**0.1798**	0.2351	0.1864	0.1994	0.2123
	Worst	**0.2254**	0.3566	0.2429	0.2411	0.2758
	Mean	**0.1980**	0.2871	0.2105	0.2118	0.2394
	Std	**0.1314**	0.3720	0.1556	0.1441	0.2173

**Table 8:** Performance of all algorithms on RNP with Cd500.

Objective Func.		HABC	PS^2^O	EGA	CCEA	PSO
*f*_*c*_	Best	**723.3412**	761.4376	931.5424	965.1634	877.0912
	Worst	**951.312**	1.024*e* + 003	1.9652*e* + 003	998.8912	1.1274*e* + 003
	Mean	**832.4521**	981.9812	1.21654*e* + 003	981.9812	983.3461
	Std	65.3476	110.2360	361.0912	**61.1653**	141.7735

*f*_*i*_	Best	**530.6258**	545.5313	539.9123	531.6734	584.4458
	Worst	**541.9812**	573.4476	568.3476	562.6423	662.9891
	Mean	546.3155	558.0246	541.6712	**545.7813**	600.2657
	Std	**4.8912**	9.2376	14.3452	8.6791	15.9824

*f*_*e*_	Best	1.0126**e** − 08	3.2345*e* − 008	4.2351*e* − 007	8.7342*e* − 007	3.6342*e* − 007
	Worst	1.7981**e** − 08	5.1852*e* − 008	9.8782*e* − 007	9.7674*e* − 007	4.4487*e* − 007
	Mean	1.4621**e** − 08	4.3186*e* − 008	7.4127*e* − 007	9.2412*e* − 007	5.1956*e* − 007
	Std	1.5431**e** − 09	6.3671*e* − 008	8.9834*e* − 007	9.8671*e* − 008	3.6423*e* − 008

*f*_*l*_	Best	6.2234**e** − 0018	7.4523412*e* − 007	4.0934*e* − 08	4.9834*e* − 07	6.4867
	Worst	4.2823**e** − 0017	5.4123*e* − 006	25.5623	0.0085	24.1438
	Mean	2.3923**e** − 0017	3.5978*e* − 006	8.0921	0.0069	10.5643
	Std	6.6574**e** − 0018	7.4123*e* − 007	8.7321	0.0067	5.8904

*f*_*m*_	Best	**0.2549**	0.3640	0.3365	0.3521	0.5051
	Worst	**0.2637**	0.3935	0.4454	0.4633	0.5322
	Mean	**0.2563**	0.3780	0.4067	0.4232	0.5168
	Std	**0.0785**	0.0412	0.3223	0.3521	0.0106

**Table 9:** Parameters of the test functions.

*f*	Dimensions	Initial Range	x*	*f*(x*)
*f*_1_	100	[−100, 100]^*D*^	[0, 0,…, 0]	0
*f*_2_	100	[−30, 30]^*D*^	[1, 1,…,1]	0
*f*_3_	100	[−5.12, 5.12]^*D*^	[0, 0,…, 0]	0
*f*_4_	100	[−500, 500]^*D*^	[420.9867,…, 420.9867]	0
*f*_5_	100	[−32.768, 32.768]^*D*^	[0, 0,…, 0]	0
*f*_6_	100	[−100, 100]^*D*^	[0, 0,…, 0]	−450
*f*_7_	100	[−100, 100]^*D*^	[0, 0,…, 0]	−450
*f*_8_	100	[−100, 100]^*D*^	[0, 0,…, 0]	390
*f*_9_	100	No bounds	[0, 0,…, 0]	−180
*f*_10_	100	[−32, 32]^*D*^	[0, 0,…, 0]	−140

## Appendix

### List of Test Functions

These benchmark functions consist of the basic benchmark continuous functions *f*_1_ ~ *f*_2_ (unimodal), *f*_3_ ~ *f*_5_ (multimodal), and the shifted and rotated functions *f*_6_–*f*_10_ (CEC2005).

*Unimodal Benchmark Function*

(1) Sphere's function:
  (A.1) $$\begin{array}{c} f_{1}\left(x\right)=\sum_{i=1}^{n}x_{i}^{2}. \end{array}$$
(2) Rosenbrock's function:
  (A.2) $$\begin{array}{c} f_{2}\left(x\right)=\sum_{i=1}^{n}100\times {\left(x_{i+1}-x_{i}^{2}\right)}^{2}+{\left(1-x_{i}\right)}^{2}. \end{array}$$

*Multimodal Benchmark Function*

(3) Rastrigrin function
  (A.3) $$\begin{array}{c} f_{5}\left(x\right)=\sum_{i=1}^{n}\left(x_{i}^{2}-10\cos\left(2\pi x_{i}\right)\right)+10. \end{array}$$
(4) Schwefel's function:
  (A.4) $$\begin{array}{c} f_{6}\left(x\right)=D*418.9829+\sum_{i=1}^{D}-x_{i}\sin\left(\sqrt{\left|x_{i}\right|}\right). \end{array}$$
(5) Ackley's function:
  (A.5) f7(x)=−20exp⁡(−0.21n∑i=1nxi2)−exp⁡(1n∑i=1ncos⁡2πxi)+20+e.

*CEC2005 Function*

(6) Shifted Sphere's function:
  (A.6) $$\begin{array}{c} f_{9}\left(x\right)=\sum_{i=1}^{D}z_{i}^{2}+f_{\text{bias}1},\;z=x-o. \end{array}$$
(7) Shifted Schwefel's Problem 1.2:
  (A.7) $$\begin{array}{c} f_{10}\left(x\right)=\sum_{i=1}^{D}{\left(\sum_{j=1}^{i}z_{j}\right)}^{2}+f_{\text{bias}},\;z=x-o. \end{array}$$
(8) Shifted Rosenbrock's function:
  (A.8) $$\begin{array}{c} f_{11}\left(x\right)=\sum_{i=1}^{D-1}\left(100{\left(z_{i}^{2}-z_{i+1}\right)}^{2}+{\left(z_{i}^{2}-1\right)}^{2}\right)+f_{\text{bias}}. \end{array}$$
(9) Shifted rotated Griewank's function without bounds:
  (A.9) $$\begin{array}{c} f_{12}\left(x\right)=\sum_{i=1}^{D}\frac{{z_{i}}^{2}}{4000}-\prod_{i=1}^{D}\cos\left(\frac{z_{i}}{\sqrt{i}}\right)+1+f_{\text{bias}}. \end{array}$$
(10) Shifted rotated Ackley's function with global optimum on bounds:
  (A.10) f13(x)=−exp⁡(−0.21D∑i=1Dzi2)−exp⁡(∑i=1Dcos⁡(2πzi))+20+e+fbias, z=(x−o)∗M.

*Parameters of the Test Functions*. The dimensions, initialization ranges, global optima *x**, and the corresponding fitness value *f*(*x**) of each function are listed in Table 9.
